# Supplementary material for: Internalization of Erythrocyte Acylpeptide Hydrolase Is Required for Asexual Replication of Plasmodium falciparum
Source: mSphere. 2019 May 8;4(3):e00077-19. doi: 10.1128/mSphere.00077-19 (PMC6506615; doi:10.1128/mSphere.00077-19)
Supplement: TABLE S1 [file mSphere.00077-19-st001.docx]

| Inhibitor | Structure | Target | Ref |
| --- | --- | --- | --- |
| AA74-1 | 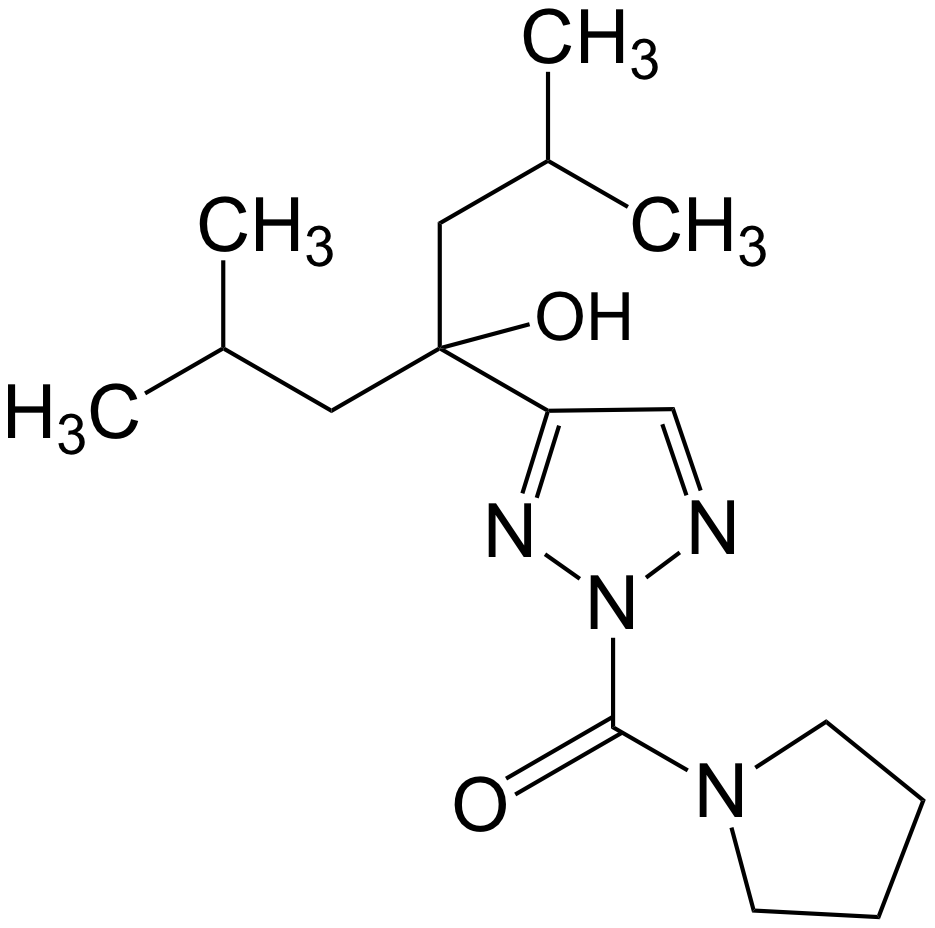 | acylpeptide hydrolase | (1) |
| WWL70 | 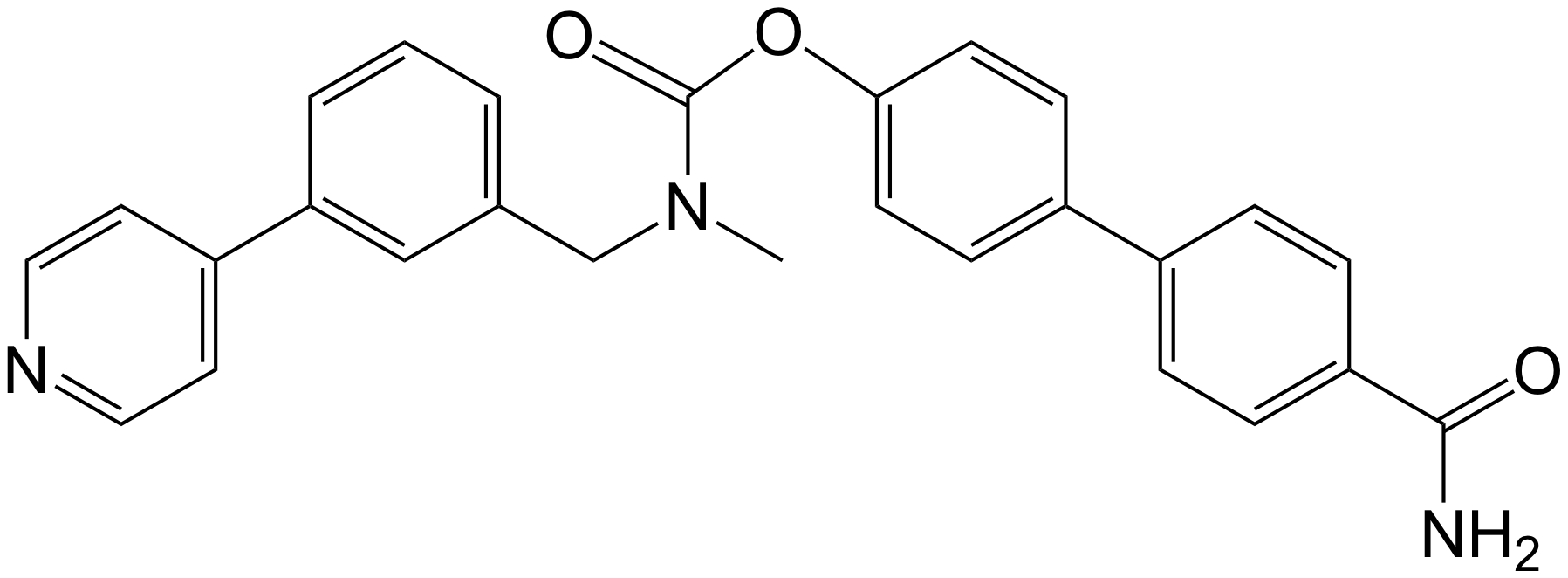 | α/β-hydrolase domain 6 | (2) |
| JW642 | 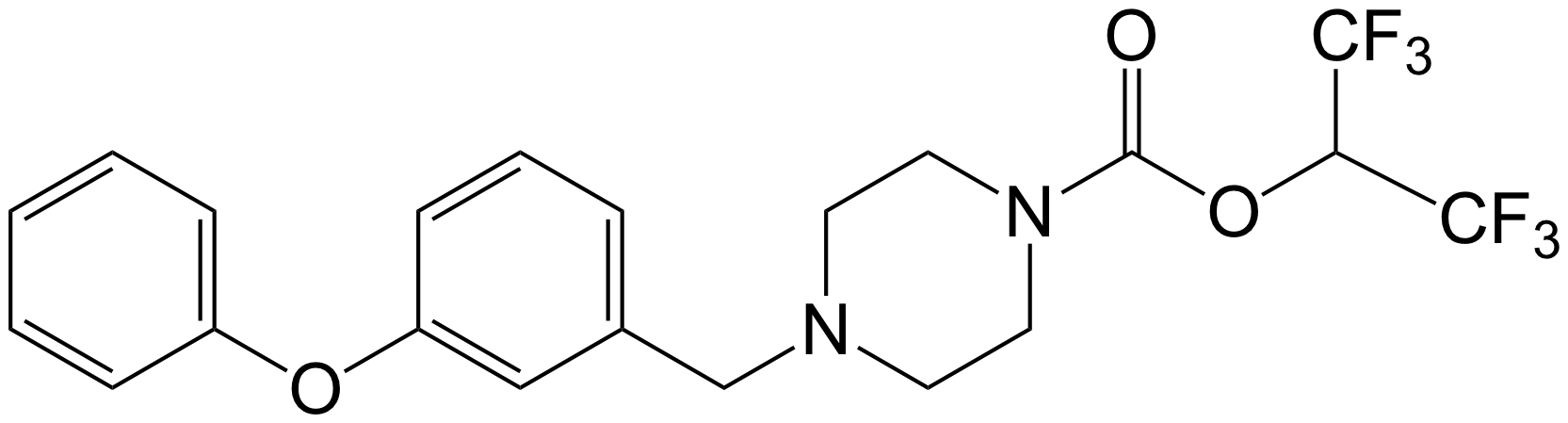 | monoacylglycerol lipase | (3) |
| KT109 | 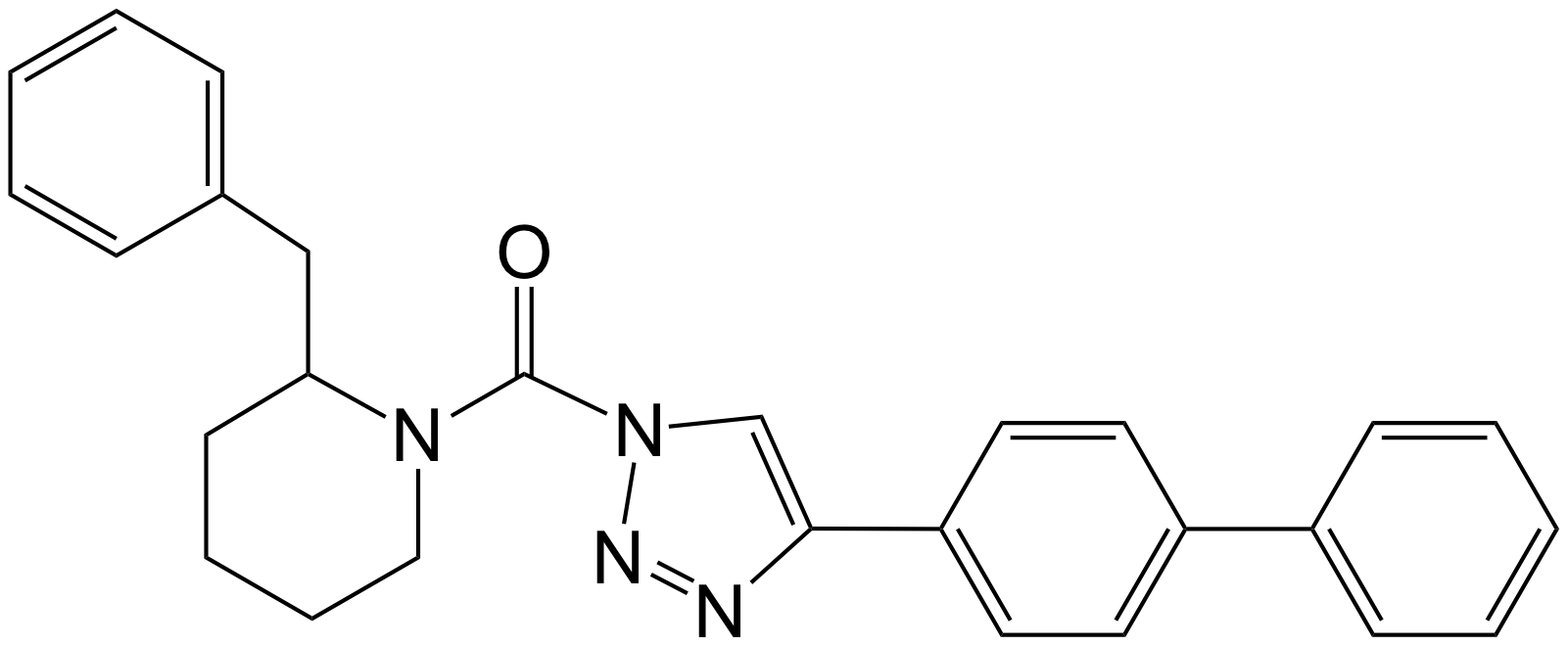 | diacylglycerol lipase | (4) |
| Orlistat | 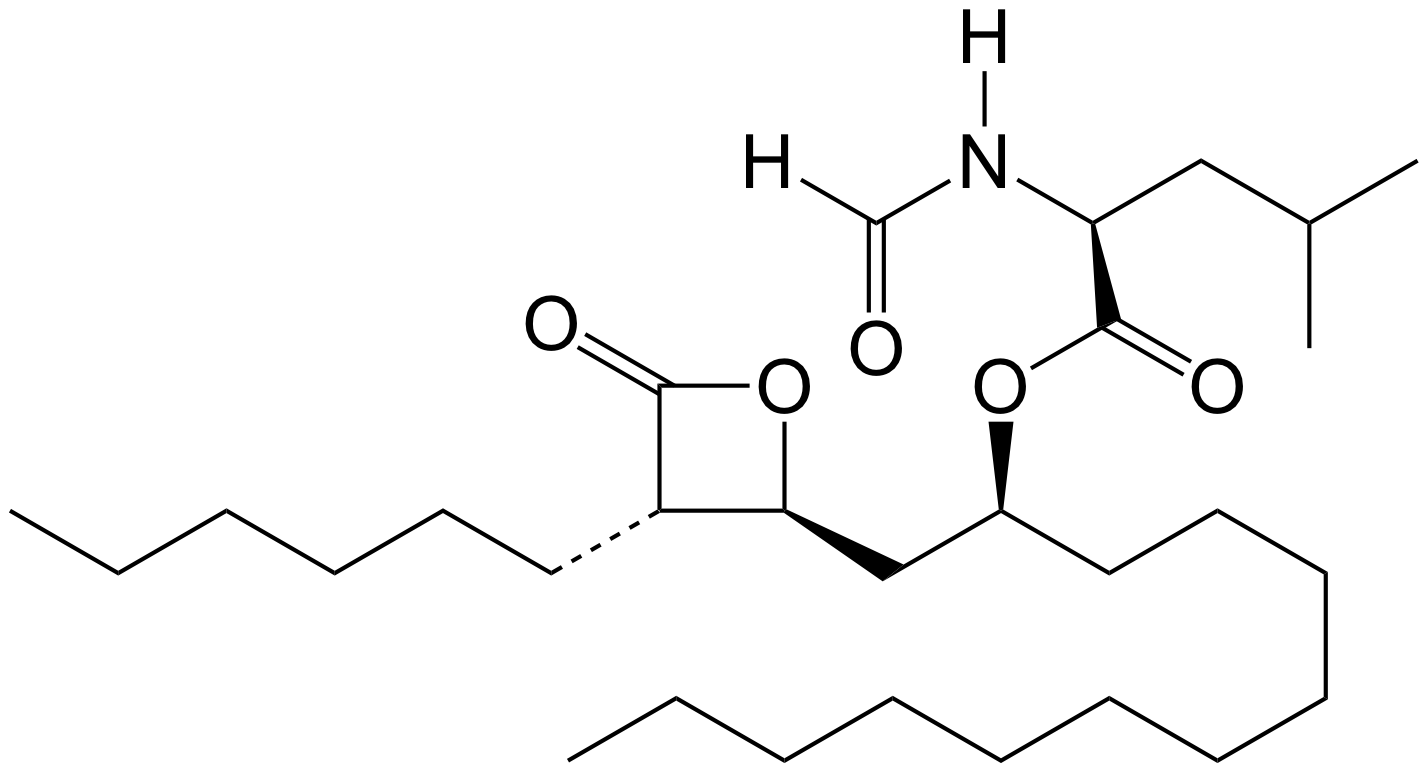 | triacylglycerol lipase | (5) |
| PF3845 | 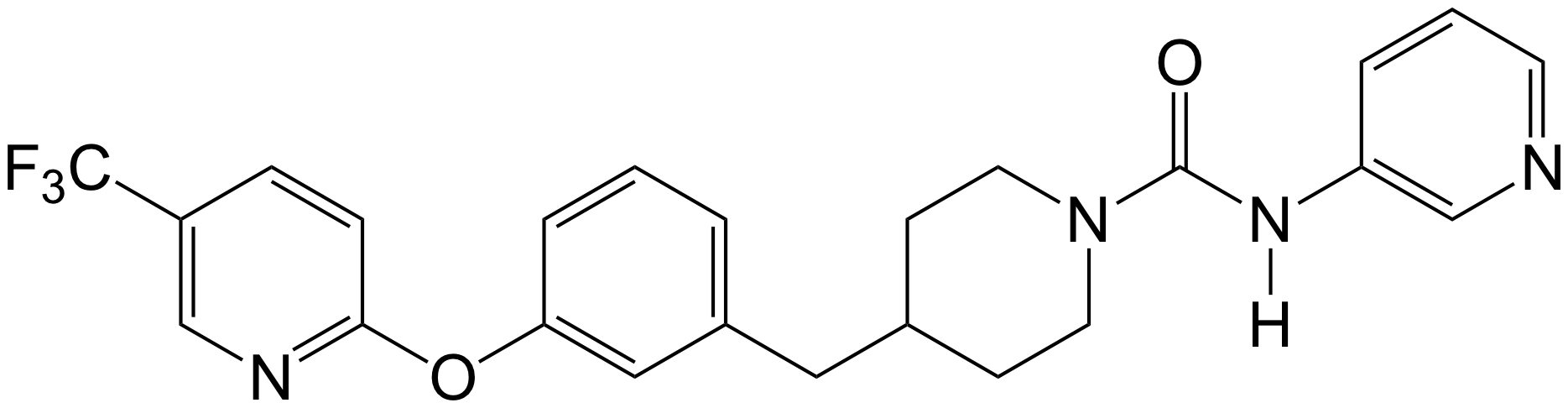 | fatty acid amide hydrolase | (6) |
| Palmostatin B | 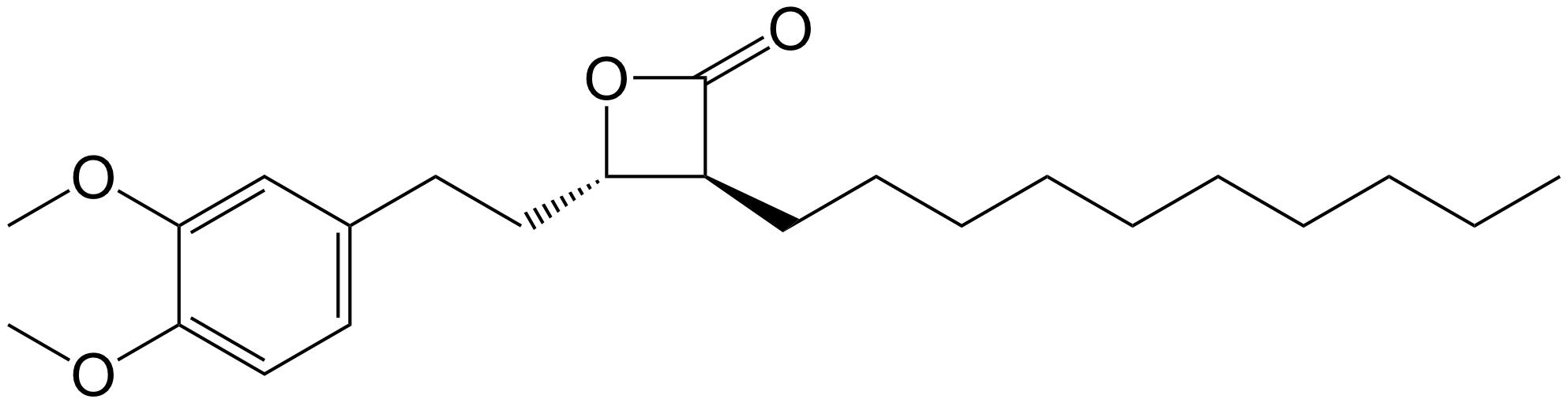 | thioesterase | (7) |

**REFERENCES**

1. Adibekian A, Martin BR, Wang C, Hsu KL, Bachovchin DA, Niessen S, Hoover H, Cravatt BF. 2011. Click-generated triazole ureas as ultrapotent, *in vivo*-active serine hydrolase inhibitors. Nat Chem Biol 7:469-478.

2. Blankman JL, Simon GM, Cravatt BF. 2007. A comprehensive profile of brain enzymes that hydrolyze the endocannabinoid 2-arachidonoylglycerol. Chem Biol 14:1347-56.

3. Chang JW, Niphakis MJ, Lum KM, Cognetta AB, 3rd, Wang C, Matthews ML, Niessen S, Buczynski MW, Parsons LH, Cravatt BF. 2012. Highly selective inhibitors of monoacylglycerol lipase bearing a reactive group that is bioisosteric with endocannabinoid substrates. Chem Biol 19:579-88.

4. Hsu KL, Tsuboi K, Adibekian A, Pugh H, Masuda K, Cravatt BF. 2012. DAGLbeta inhibition perturbs a lipid network involved in macrophage inflammatory responses. Nat Chem Biol 8:999-1007.

5. Hadvary P, Lengsfeld H, Wolfer H. 1988. Inhibition of pancreatic lipase *in vitro* by the covalent inhibitor tetrahydrolipstatin. Biochem J 256:357-61.

6. Ahn K, Johnson DS, Mileni M, Beidler D, Long JZ, McKinney MK, Weerapana E, Sadagopan N, Liimatta M, Smith SE, Lazerwith S, Stiff C, Kamtekar S, Bhattacharya K, Zhang Y, Swaney S, Van Becelaere K, Stevens RC, Cravatt BF. 2009. Discovery and characterization of a highly selective FAAH inhibitor that reduces inflammatory pain. Chem Biol 16:411-20.

7. Dekker FJ, Rocks O, Vartak N, Menninger S, Hedberg C, Balamurugan R, Wetzel S, Renner S, Gerauer M, Scholermann B, Rusch M, Kramer JW, Rauh D, Coates GW, Brunsveld L, Bastiaens PI, Waldmann H. 2010. Small-molecule inhibition of APT1 affects Ras localization and signaling. Nat Chem Biol 6:449-56.
