# Supplementary figures and images for: Internalization of Erythrocyte Acylpeptide Hydrolase Is Required for Asexual Replication of Plasmodium falciparum
Source: mSphere. 2019 May 8;4(3):e00077-19. doi: 10.1128/mSphere.00077-19 (PMC6506615; doi:10.1128/mSphere.00077-19)

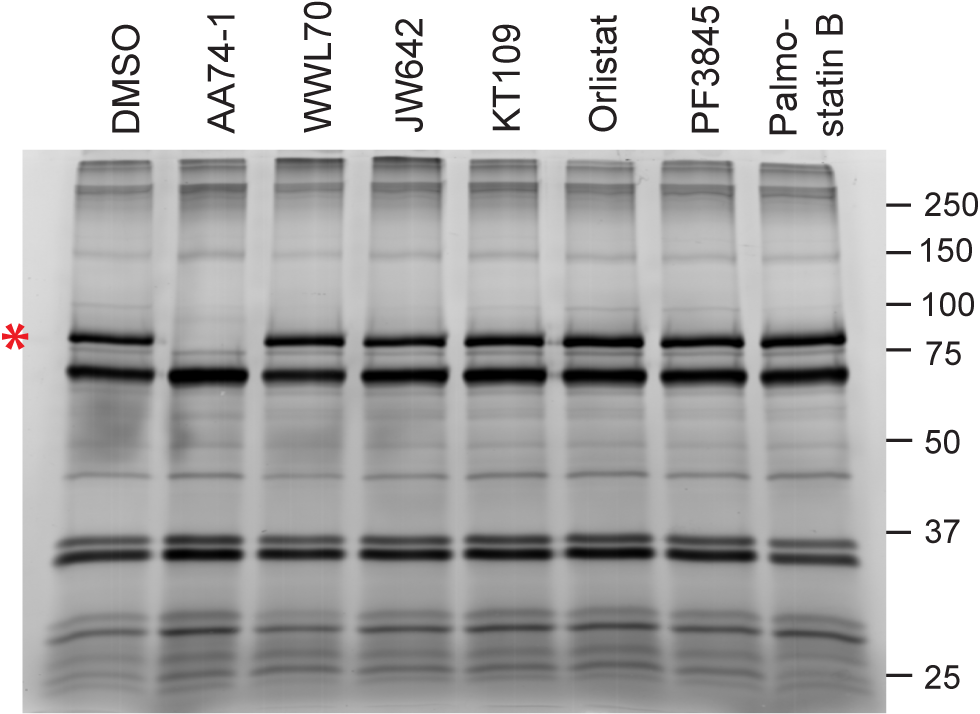

Supplement: FIG S1 [file mSphere.00077-19-sf001.tif]

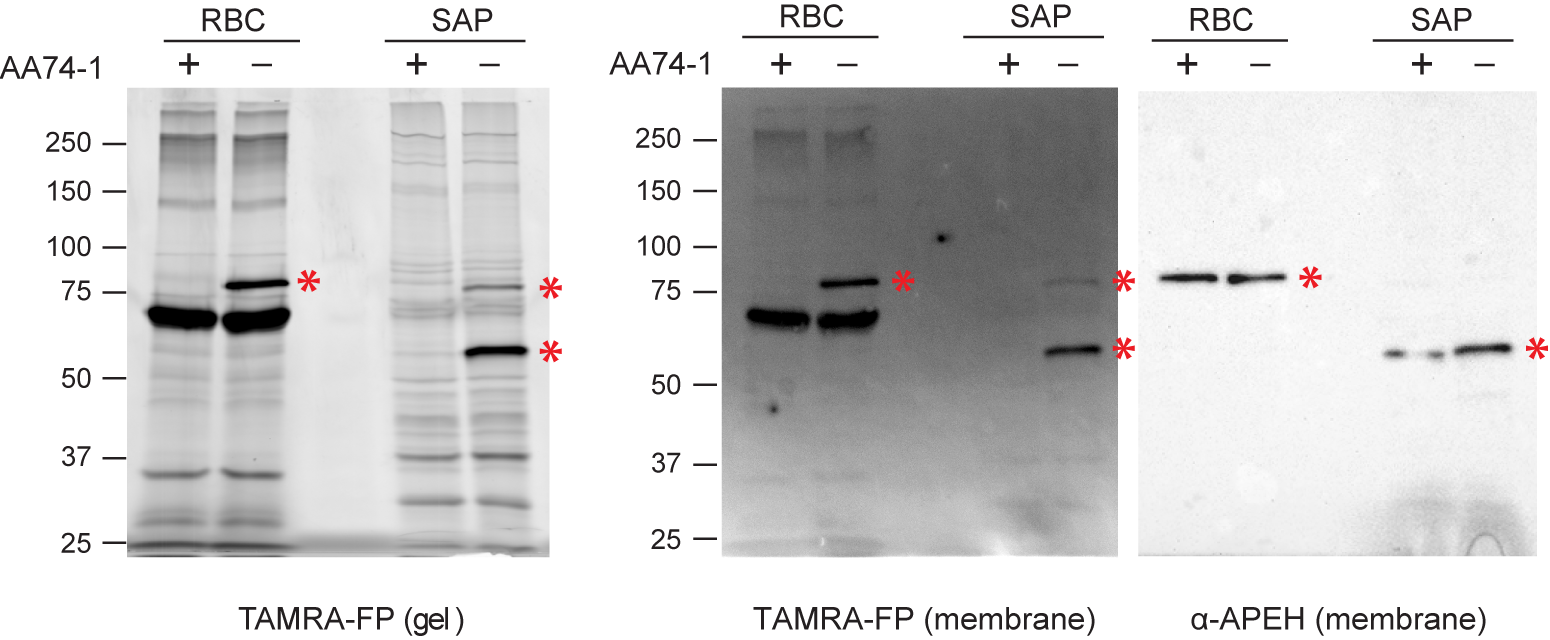

Supplement: FIG S2 [file mSphere.00077-19-sf002.tif]

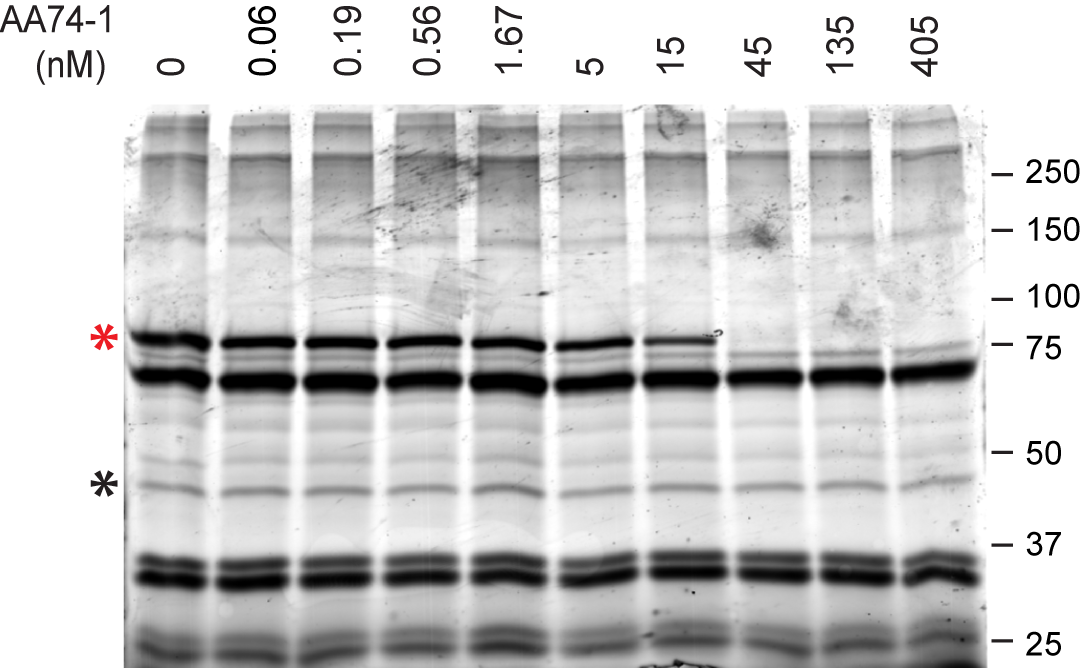

Supplement: FIG S3 [file mSphere.00077-19-sf003.tif]

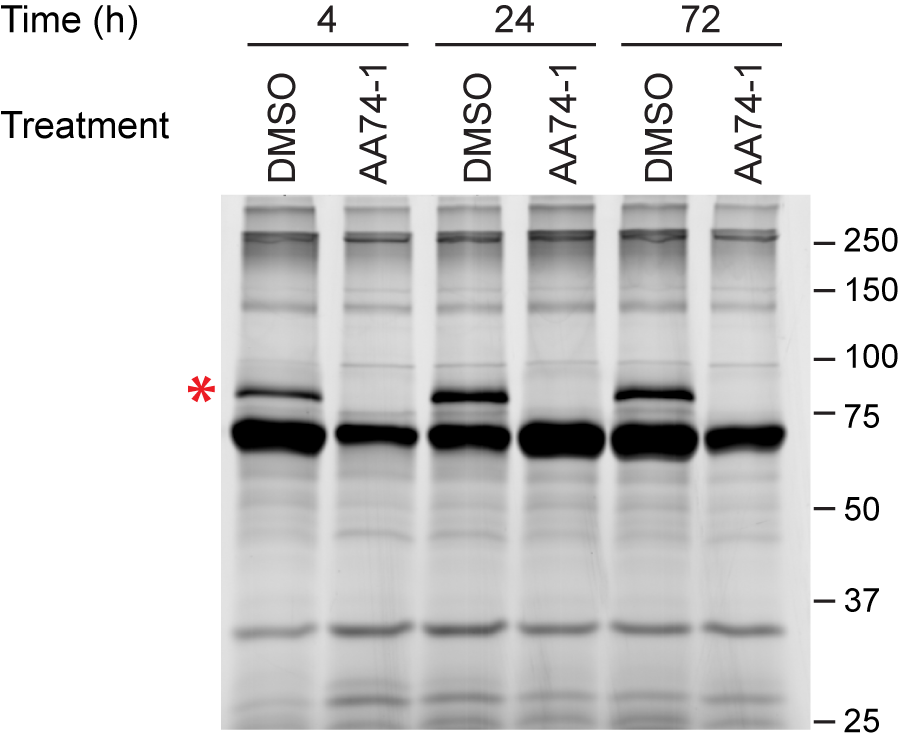

Supplement: FIG S4 [file mSphere.00077-19-sf004.tif]
